# Dynamic Gene-Resource Landscape Management of Norway Spruce: Combining Utilization and Conservation

**DOI:** 10.3389/fpls.2017.01810

**Published:** 2017-10-18

**Authors:** Milan Lstibůrek, Yousry A. El-Kassaby, Tore Skrøppa, Gary R. Hodge, Jørn H. Sønstebø, Arne Steffenrem

**Affiliations:** ^1^Faculty of Forestry and Wood Sciences, Czech University of Life Sciences Prague, Prague, Czechia; ^2^Faculty of Forestry, The University of British Columbia, Vancouver, BC, Canada; ^3^Norwegian Institute of Bioeconomy Research, Ås, Norway; ^4^Camcore, Department of Forestry and Environmental Resources, North Carolina State University, Raleigh, NC, United States

**Keywords:** gene diversity, *in situ* selection, climate change, adaptation, tree improvement, DNA markers

## Abstract

Traditional gene-resource management programs for forest trees are long-term endeavors requiring sustained organizational commitment covering extensive landscapes. While successful in maintaining adaptation, genetic diversity and capturing traditional growth attributes gains, these programs are dependent on rigid methods requiring elaborate mating schemes, thus making them slow in coping with climate change challenges. Here, we review the significance of Norway spruce in the boreal region and its current management practices. Next, we discuss opportunities offered by novel technologies and, with the use of computer simulations, we propose and evaluate a dynamic landscape gene-resource management in Norway. Our suggested long-term management approach capitalizes on: (1) existing afforestation activities, natural crosses, and DNA-based pedigree assembly to create structured pedigree for evaluation, thus traditional laborious control crosses are avoided and (2) landscape level genetic evaluation, rather than localized traditional progeny trials, allowing for screening of adapted individuals across multiple environmental gradients under changing climate. These advantages lead to greater genetic response to selection in adaptive traits without the traditional breeding and testing scheme, facilitating conservation of genetic resources within the breeding population of the most important forest tree species in Norway. The use of *in situ* selection from proven material exposed to realistic conditions over vast territories has not been conducted in forestry before. Our proposed approach is in contrast to worldwide current programs, where genetic evaluation is constrained by the range of environments where testing is conducted, which may be insufficient to capture the broad environmental variation necessary to tackle adaptation under changing climate.

## Significance of Norway Spruce in the Boreal Region

Bio-products from forest trees have always been important for societies in boreal regions, and will continue to be important for a successful transfer to a bio-based economy. Production forestry in this region is typically based on Norway spruce (*Picea abies* L. Karst) and Scots pine (*Pinus sylvestris* L.) timber resources across the landscape, utilizing areas traditionally not suitable for effective production of other crops. The landscape delivers important ecosystem services, non-timber values, and is of fundamental importance for the biodiversity. Norway spruce is the most widespread and economically important conifer tree in Europe.

In Norway, regeneration of harvested forest sites is mainly done through planting with Norway spruce seedlings originating from conventional tree breeding programs. These programs follow a systematic recurrent selection breeding scheme, with long-term repeated cycles of selection, breeding and testing (White et al., 2007). The main goal of these programs is to deliver forest reproductive materials (FRM) with wide adaptability for meeting not only superior wood quality and productivity needs, but also unexpected contingencies such as biotic and abiotic threats exacerbated by climatic change (Edvardsen et al., 2010; Jansson et al., 2016). The development of FRM must satisfy multiple stakeholders, including forest owners, industrial enterprises, and societal needs for maximizing economic revenue, CO_2_ sequestration, and adaptation to climate change that is progressing with unprecedented speed and magnitude. While forest tree species are very genetically diverse, they exhibit extensive phenotypic plasticity, thus the breadth and depth of FRM’s genetic diversity are of vital importance for maintaining populations’ evolutionary potential, a prerequisite for dealing with unforeseen contingencies (Alfaro et al., 2014). Both neutral and adaptive diversity of forest trees are spatially distributed across the landscape, so conservation planning, assessment, and monitoring of forest genetic resources (FGR) must take place at the same scale (Lefèvre et al., 2013 and references therein). Thus, a deliberate development and management of FGR are important to maintaining diverse ecosystems, species adaptive potential, and to safeguard the economy of the boreal region forest enterprise.

## Evolutionary Perspectives and Genomics

The genetic structure of forest tree populations is influenced jointly by the effects of selection, mutation, migration and random genetic drift (Hartl and Clark, 2007). Norway spruce reached its western distribution in Norway during the Holocene, with ancestry in an Eastern-European refugium near Moscow (Tollefsrud et al., 2008), and possibly a western but still unknown refugium (Parducci et al., 2012a,b). Since their first settlements, humans have been influencing the genetic composition of forest tree species, but the major influence has been industrial wood consumption which led to a rationale for plantation forestry, i.e., trees became a plantation crop (Savill et al., 1997; Myking et al., 2016). In Europe, extensive forest management has evolved in most countries and commercial tree species, such as Norway spruce, are major resources in the bio-economy.

Norway spruce’s genome is the first sequenced conifer and is considered to be among the largest genomes of all organisms with 20–30 gigabases (Nystedt et al., 2013). The genome sequence revealed unprecedented complexity with high-copy repeat content and only a small functional component (i.e., genes). Both the large complex genome size and the low density of genes make genome wide analyses of Norway spruce challenging. This required the development of genome reduction alternative methods for assessing their genome wide variation, including exome capture (Neves et al., 2013) as well as non-ordered sequencing through genotyping by sequencing (Chen et al., 2013). Along with the availability of the species genome sequence, these methods made it possible to conduct genome wide association analyses for inclusion in breeding programs (Ingvarsson and Street, 2011).

## Current Management of Norway Spruce’s Gene Resources

The first cycle of the Norwegian breeding program started in the 1970’s with progeny testing of phenotypically selected trees (plus-trees, F_0_) from mature natural grown stands in Norway. Since then, about 3,500 plus-trees have been tested for performance of their progeny (F_1_) in multiple field trials. Among the F_1_, a superior population is selected as the basis for long-term breeding, structured in 23 sublines of approximately 50 individuals based on adaptive characteristics (Edvardsen et al., 2010). To minimize loss of genetic diversity, a balanced mating and selection scheme is proposed to ensure the representation of the founders in subsequent generations (Danell, 1991; Haapanen and Mikola, 2008). Although Norway spruce is known for its high degree of phenotypic plasticity, it is susceptible to damages from frost events early in the growing season (Skrøppa and Steffenrem, 2015; Chen et al., 2017) which are predicted to become more frequent with climate change (Langvall, 2011). Therefore, population adaptation to climate change is considered an important breeding objective, along with better growth rate and wood quality. Height growth is the backbone of initial selection as it is a proxy for fitness. The narrow-sense heritability (*h*^2^) typically range from low (∼0.15) for growth traits, intermediate (∼0.50) for quality traits, to high (>0.60) for adaptive traits such as phenology (Skrøppa and Steffenrem, 2015; Steffenrem et al., 2016). It should be stated, however, that the conventional breeding approaches are facing a multitude of limitations and challenges, mainly related to the organizational long-term financial commitment and the time required for completing one cycle of selection, breeding and testing. Thus, novel and innovative approaches are needed to assist in overcoming these limitations.

## Opportunities of Novel Concepts and Technologies

Forest tree breeding programs are historically influenced by advancements in agriculture, animal, and crop breeding (White et al., 2007). Recently, the success of genomic selection (GS; Meuwissen et al., 2001) in the dairy industry has generated great excitement in forest tree breeding community, primarily due to the opportunity to substantially reduce the breeding cycle time and increase selection differential, which potentially will increase the genetic gain per unit of time, effort, and cost (Grattapaglia and Resende, 2011). GS is based on a single major assumption, mainly the existence of linkage disequilibrium (LD) between genetic markers and the causal genes underlying target traits, thus members of the breeding population need to be genotyped for an exceedingly large number of markers to overcome the fast LD decay that is characteristic of outcrossing species such as forest trees (i.e., deep sequencing is required to secure LD between markers (SNPs) and causal genes). Most forestry target traits are complex in nature (i.e., follow Fisher’s infinitesimal model), are expressed at advanced life stages, and are characterized by low heritability and pronounced genotype × environment interaction, thus posing challenges to GS implementation. To overcome GS challenges, larger training populations and extensive marker densities are required (Hayes et al., 2009). Additionally, while GS has the potential to overcome some of the conventional breeding drawbacks, the fact that it is population-specific requires multiple GS programs for different breeding zones / populations. Furthermore, the forestry production system of genetically improved stock is somewhat different from their agriculture counterparts, as foresters sometimes intentionally sacrifice gain for the maintenance of genetic diversity, needed for future selection cycles and securing adaptation in planted forests (Lindgren and Mullin, 1997).

While the establishment, maintenance, and phenotyping of progeny test trials constitute a major cost in traditional breeding programs, the added costs associated with the existing tree improvement infrastructure and competence needed to support the program are hardly considered when these programs are financially evaluated. As an alternative to the conventional breeding approaches, El-Kassaby and Lstibůrek (2009) introduced the concept of “Breeding-without-Breeding” (BwB) that avoids the dependency on structured pedigree and relies on simple field testing to attain genetic gains comparable to that captured from conventional programs. In their method, structured pedigree is assembled through paternity assignment rather than that created from conventional controlled crosses among the breeding population’s parents. This is accomplished using a subset of highly polymorphic markers for fingerprinting offspring produced from random open-pollinated matings and the assembled pedigree is subsequently used in the genetic evaluation. Genetic evaluation is conducted using “standard” quantitative genetics analytical methods such as the REML-BLUP approach (Henderson, 1976). This concept utility for estimating parental and offspring populations breeding values was demonstrated in western larch and the results were identical to those produced from conventional breeding method (El-Kassaby et al., 2011). To further reduce the cost, genotyping and phenotyping efforts are restricted to two groups of individuals representing a reduced set of top-tier individuals and a random sample of the testing population and this approach has produced results comparable to conventional breeding methods (Lstibůrek et al., 2015). In conclusion, the BwB concept is thoroughly investigated and factors such as variable parental gametic contributions and pollen flow from external sources have been assessed and both genetic gain and diversity were sufficiently robust (Lstibůrek et al., 2011, 2012, 2015).

Phenotyping of trees has been identified as a major bottleneck as it is costly and often tedious. The development of alternative fast and reasonably inexpensive phenotyping methods would be of great value to forest managers and tree breeders. Remote sensing techniques for estimation tree heights and canopy characteristics, such as airborne laser scanning (e.g., Solberg et al., 2006) or 3D imagery (Birdal et al., 2017), have the potential to considerably reduce phenotyping costs. For example, a pilot study conducted by Steffenrem et al. (2014) has shown that a 3D digital surface model, obtained by unmanned aerial vehicle (UAV), subtracted from a terrain models obtained from laser scanning, can be used for progeny trials’ tree height estimation even in rugged terrain. High resolution terrain models from laser scanning are now becoming available from regional mapping projects, thus more effective phenotyping of larger populations across wider environmental gradients is expected to further simplify attributes assessment.

## Perspectives on the Long-Term Management of Genetic Resources

To increase the cost-efficiency of marker-based approaches to FGR management, Muranty et al. (2014) and others suggested scaling down the census size of the candidate populations while maintaining high genetic variability. We feel that this approach is contrary to the broader goal aiming at optimal gene resource management, and since we face unpredictable climate change and other risks, scaling up population sizes from current standards seems more appropriate. It is well understood that the likelihood of preserving rare alleles is proportional to the population size (Allendorf, 1986), and such rare alleles may be important under future unknown climate or other contingency scenarios. Thus, significantly larger populations should be screened across multiple and wide environmental gradients. In this way, foresters would emphasize fitness value, and natural selection should be promoted (i.e., active *in situ* management strategy). Therefore, we propose to work with large populations, rely on natural crosses (and natural selection imposed through the reproduction and plantations’ development), and then evaluate very large candidate populations on a landscape basis, rather than dealing with a limited number of progeny test trials. This is also compatible with the genotype × environment interactions commonly observed in forest tree trials and the generally unpredictable effects of long-term selection on genetic covariance among multiple traits (Namkoong, 1979).

## A New Approach: Overview

The proposed approach is summarized here, under the assumptions that four sublines will form the base for selection needed to establish a new seed orchard within a breeding zone in Norway. A subline is a subset of the breeding population managed independently from other subsets to minimize inbreeding and the build-up of coancestry. An identical approach would be followed in each of the four sublines for a given breeding zone.

For each subline, a breeding arboretum is established as a “breeding population.” Following natural crossing (open-pollination), conventional forest plantations are established from seed collected in the arboretum, and managed as regular commercial plantations. We assume that all the phenotyping, genetic evaluation and selection for the gene conservation and tree breeding program is performed in these plantations. A typical plantation of Norway spruce is assumed to be 3 hectares in size with 6,000 surviving trees when the plantation reaches an appropriate age for evaluation and selection. First, a Random subpopulation of size = *N*_R_ is measured (phenotyped) in the plantations. In this step, the breeder simply selects uniform, well-stocked sections of the plantations, and measures contiguous blocks of trees as in a normal progeny trial. Second, a very large population residing in established plantations is screened to identify a small Top Phenotype subpopulation of size = *N*_T_. Based on phenotypic assessment, these trees are the top candidates for selection. The idea is to use UAV to screen tens of thousands of trees and make an identification of candidates. Molecular marker analysis and pedigree reconstruction will be done on both *N*_R_ and *N*_T_ trees, respectively. Finally, all phenotypic data are utilized to estimate genetic values for all trees (*N*_R_ + *N*_T_), using the reconstructed pedigree data to estimate genetic variance components and calculate Best Linear Unbiased Predictions (BLUPs).

## Quantitative Evaluation of the Proposed Approach

Computer simulations were done to compare the genetic gain from the proposed approach with a conventional tree improvement program. All simulations were done following the approach presented by Lstibůrek et al. (2015), which will be briefly summarized here. A timeline for the compared scenarios is given in **Table 1**.

**Table 1:** Timeline for the progress of a breeding cycle for the proposed BwB and traditional strategy.

Year	Activity	BwB strategy	Traditional strategy
0	Mating	50 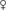 × open pollination	25 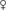 × 25 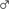
2	Planting	1 SITE = 6,000	25 families
		4 SITES = 24,000	40 trees family^-1^
		8 SITES = 48,000	4 progeny trials
		30 SITES = 180,000	∼4,000 trees
17	Evaluation	*N*_R_ = 1,800	4,000 trees
		*N*_T_ = 600	(minus mortality)
	Selection from subline	5 for a seed orchard	
		20 for the breeding arboretum	

*This makes the population for one of the four sublines within breeding zone. Total numbers of trees in the BwB experiments (N_*BwB*_) are given for 1, 4, 8 and 30 sites. *N*_*R*_ and *N*_*T*_ is the random and top phenotype subpopulations, respectively.*

A single subline was modeled with constant population size of 50 individuals. The breeding objective was assumed to be height growth at 15 years, and we assumed typical genetic parameters for *P. abies* in Norway (*h*^2^ = 0.15, with dominance variance and additive by environment interaction variance both assumed to be equal to one half of the additive genetic variance).

For the proposed BwB strategy, natural open-pollinated mating between the sub-line**’s** 50 parents was assumed with the actual variable gametic contributions typical for Norway spruce (J. Sønstebø, personal communication, Norwegian Institute of Bioeconomy Research, 2017). The Random subpopulation was held to a constant size *N*_R_ = 1,800 distributed evenly across all test sites (i.e., plantations of 6,000 trees), with the number of test sites ranging from *N*_SITES_ = 1 to 30. Thus, the total number of trees in the experiment *N*_BwB_ was *N*_SITES_ × 6,000. The Top Phenotype subpopulation was also held to a constant size of *N*_T_ = 600, distributed across test sites, wherever the best phenotypes were found. Subpopulation sizes of *N*_R_ = 1,800 and *N*_T_ = 600 resulted in sufficient genetic gains from selection across varying sizes of *N*_BwB_, and for different sizes of the selected population, while minimizing costs associated with collecting phenotypic data and lab genotyping.

For a comparison with the BwB strategy, we assumed a conventional tree improvement program with full-sib crosses (FS). Under the FS scheme, 25 FS (i.e., single-pair mating) were generated among the 50 parents with 160 trees per each full-sib family. Progeny trials were established across 4 sites within the breeding zone (40 trees per family per site). The total number of trees in the experiment was 4,000.

For both the BwB and the FS strategy, simulated phenotypic and genetic data was generated using the assumed genetic parameters. Genetic evaluation was conducted using a REML & BLUP analysis with ASReml software (Gilmour et al., 2009) with the animal model (i.e., an individual tree model). The full linear model included additive genetic effects, additive by environment effects, and dominance genetic effects.

Following the genetic evaluation, selection of the top offspring was done for a seed orchard with *N*_e_ (effective population size) = 20 unrelated parents, and a next-generation breeding arboretum of 80 unrelated selections. With four sublines in a breeding zone, this meant that from a given subline, we made *N*_e_ = 5 unrelated selections for the seed orchard, and *N*_e_ = 20 unrelated selections for the breeding arboretum. Selection of the best set of offspring for these new populations was done using a linear optimization algorithm which is fully described in Lstibůrek et al. (2015). This algorithm maximizes the genetic gain from selection for a given *N*_e_, while ensuring that there is no relatedness among the selected trees (to avoid the impact of inbreeding depression on adaptive and other traits).

**FIGURE 1:**
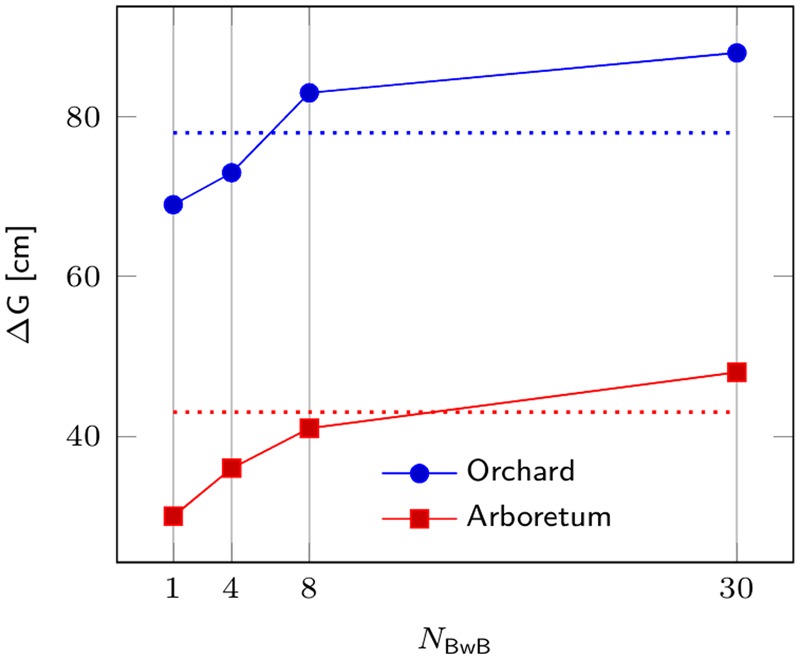
Comparison of genetic response from a BwB strategy and a conventional tree breeding strategy for Norway spruce. Genetic response ΔG is depicted on *Y*-axis as a function of the number of stands in the BwB strategy (*N*_BwB_). Blue and red lines refer to gains for the Seed Orchard (*N*_e_ = 20) and Breeding Arboretum (*N*_e_ = 80), respectively, developed from four sublines for a given breeding zone. Dotted lines depict the corresponding ΔG for a conventional full-sib breeding strategy.

Based on simulation results (**Figure 1**), we believe the proposed BwB approach is very competitive to the traditional forest tree selective breeding programs. For example, for the Seed Orchard population, gain from the BwB approach surpasses conventional programs with *N*_SITES_ > 6. The BwB strategy requires a Random subpopulation of 1,800 trees (evenly distributed across 6 or more commercial plantations) to be phenotyped and genotyped. An additional 600 Top Phenotype trees are identified by cost-effective UAV, and these trees are also phenotyped and genotyped. This approach will produce genetic gains greater than or equal to conventional breeding approaches, and could be more resource efficient due to reduced cost and time components.

We further investigated the sensitivity of ΔG with respect to the key parameters in the current study. Significant incremental response to selection of the BwB strategy was observed for the studied values of *N*_BwB_ (**Figure 1**). A *t*-test was conducted to compare genetic response to selection between the two respective strategies, i.e., BwB and conventional full-sib strategy at each value of *N*_BwB_. All differences were statistically significant (*P* ≤ 0.0001), except for the respective gains for the arboretum population at *N*_BwB_ = 8. As Lstibůrek et al. (2015) highlighted, both strategies produce moderately high correlation of the true and predicted genetic values for progeny selection. Thus, the observed advantage of the BwB (larger *N*_BwB_) over the full-sib alternative is due to the larger within-family selection intensity (larger size of the candidate population). Results were robust to additional increments in *N*_R_, as the current size of 1,800 was sufficient to variance decomposition and prediction of the respective mid-parental additive genetic values, thus further increase in the *N*_R_ parameter produced non-significant ΔG of the BwB strategy (data not shown). Lstibůrek et al. (2015) provide further theoretical discussion on the topic and on the sensitivity of the BwB scenario to additional parameters.

## Conclusion and Future Perspectives

The main features of the proposed gene-resource management could be highlighted as: (1) natural open-pollinated crosses, thus natural selection is targeted at adaptive traits, (2) genetic testing, screening, and evaluation are performed on the landscape level, recognizing the importance of adaptive traits and their interaction with environmental effects and their spatial and temporal change, (3) selection optimally utilizing available information, highlighting the importance of genetic diversity, yet providing sufficient genetic gains in productive traits mainly due to the expansion of the candidate population, thus increasing the within-family selection intensity, and (4) the strategy is logistically simple and reduces complex and expensive operations (i.e., avoidance of control crosses and establishment of field trials). After selections, the forest plantations will be managed for production of timber until the economic rotation age is attained, and therefore remain as a possible base for new selections and reservoirs of genetic variability for at least 50 additional years.

We believe that once high-density SNP chips for Norway spruce become available, combined with further genotyping cost reductions, it will be possible to replace (within the identical BwB approach) the BLUP-based evaluation by the genomic (GBLUP) alternative (utilizing the realized relationships). This could further enhance the BwB gains due to: (1) accounting for historical relationships, (2) more efficient separation of non-additive genetic variance components, and (3) utilization of the Mendelian sampling term (El-Dien et al., 2016), while still maintaining the same benefits of the BwB.

The proposed approach could be applicable to any species world-wide, where the adaptive response to climate change is becoming a critical component to human population growth (wood demand) and where resources to implement traditional breeding are limited.

## Author Contributions

ML and AS conceived the project and supervised the manuscript. ML, YE-K, TS, GH, JS, and AS drafted the manuscript and contributed to discussions. ML and AS conducted computer simulations.

## Conflict of Interest Statement

The authors declare that the research was conducted in the absence of any commercial or financial relationships that could be construed as a potential conflict of interest.
